# Discovery of the SHP2 allosteric inhibitor 2-((3R,4R)-4-amino-3-methyl-2-oxa-8-azaspiro[4.5]decan-8-yl)-5-(2,3-dichlorophenyl)-3-methylpyrrolo[2,1-f][1,2,4] triazin-4(3H)-one

**DOI:** 10.1080/14756366.2022.2151594

**Published:** 2022-12-08

**Authors:** Yanmei Luo, Jin Li, Yuliang Zong, Mengxin Sun, Wan Zheng, Jiapeng Zhu, Liu Liu, Bing Liu

**Affiliations:** a School of Medicine & Holistic Integrative Medicine, Nanjing University of Chinese Medicine, Nanjing, China; b Division of Medicinal Chemistry, PharmaBlock Sciences (Nanjing), Inc., Nanjing, China; c Jiangsu Key Laboratory for Pharmacology and Safety Evaluation of Chinese Materia Medica, School of Pharmacy, Nanjing University of Chinese Medicine, Nanjing, China

**Keywords:** SHP2, allosteric inhibitor, X-ray crystallography

## Abstract

The non-receptor protein tyrosine phosphatase (PTP) SHP2 encoded by the PTPN11 gene is a critical regulator in a number of cellular signalling processes and pathways, including the MAPK and the immune-inhibitory programmed cell death PD-L1/PD-1 pathway. Hyperactivation and inactivation of SHP2 is of great therapeutic interest for its association with multiple developmental disorders and cancer-related diseases. In this work, we characterised a potent SHP2 allosteric inhibitor 2-((3 R,4R)-4-amino-3-methyl-2-oxa-8-azaspiro[4.5]decan-8-yl)-5-(2,3-dichlorophenyl)-3-methylpyrrolo[2,1-f][1,2,4]triazin-4(3H)-one (PB17-026-01) by using structure-based design. To study the structure–activity relationship, we compared co-crystal structures of SHP2 bound with PB17-026-01 and its analogue compound PB17-036-01, which is ∼20-fold less active than PB17-026-01, revealing that both of the compounds are bound to SHP2 in the allosteric binding pocket and PB17-026-01 forms more polar contacts with its terminal group. Overall, our results provide new insights into the modes of action of allosteric SHP2 inhibitor and a guide for the design of SHP2 allosteric inhibitor.

## Introduction

The non-receptor protein tyrosine phosphatase (PTP) SHP2 encoded by the PTPN11 gene, also named PTP1D or PTP-2C, is the first reported proto oncoprotein of human PTP family that is widely expressed in various adult cells and tissues.^1^ The structure of SHP2 consists of three domains: the Src homology (SH) regions, one catalytic PTP domain (residues 247–517), and one C-terminal tail with tyrosyl phosphorylation sites (Y542 and Y580) as well as a proline-rich motif.^2^ The SH regions denoted as N-SH2 (residues 6–104) and C-SH2 (residues 112–216) are recognition elements to bind the phosphorylated tyrosine protein sequences. X-ray structures demonstrate that the basal state SHP2 adopts an auto-inhibited conformation, of which the “backside loop” of the N-SH2 domain intramolecularly interacts with the catalytic surface of the PTP domain at the N-SH2: PTP interface to block access of the substrate to the catalytic site. The C-SH2 domain acts as a linker connecting the neighbour N-SH2 and PTP sites. However, neither of them shares a significant interface with C-SH2 domain. The activity of SHP2 is regulated by a mechanism similar to the “molecular switch”. Receptor tyrosine kinases (RTKs) are first activated by tyrosine-phosphorylated upstream signal factors (such as IL-2, IL-6, IFN-alpha, and EGF), leading to the distortion of the N-SH2 domain from the PTP domain, which makes the catalytic site accessible to substrates and changes SHP2 to the “on” state. Consequently, RAS is directly dephosphorylated and associated with the effector protein RAF. Therefore, SHP2 acts as a mediator in cell signal transduction downstream of various RTKs, including the RAS/MAPK/ERK, PI3K/AKT, mTOR, and JAK/STAT pathways.^3^ Recently, this ubiquitously expressed enzyme is found to be required for full and sustained participation in the immune-inhibitory reactions including programmed cell death-1 (PD-1) to modulate the activation of T cells. Thus, SHP2 is a fascinating target in immuno-oncology.^4^^,^^5^ Taken together, SHP2 is involved in the regulation of diverse phase in the cell life such as proliferation, survival, differentiation, migration, and apoptosis. Hyperactivation of SHP2 caused by PTPN11 germline or somatic mutations contributes to oncogenesis and underlies developmental disorders. Active mutations of SHP2 have been identified in Noonan syndrome,^6^ juvenile myelomonocytic leukaemia,^7^ B-cell acute lymphoblastic leukaemia, myelodysplastic syndrome, acute myeloid leukaemia, and various solid tumours including lung adenocarcinoma, colon cancer, breast cancers, gastric cancer and glioblastoma, neuroblastoma, melanoma, and hepatocellular carcinoma.^8^ In LEOPARD syndrome, mutation is located in the SHP2 catalytic domain, which abolishes the SHP2 phosphatase activity.^9^ SHP2 is a promising therapeutic target for its functions of regulating the aforementioned signalling pathways and the potential link between diseases and SHP2 mutations.

As an excellent multi-disease target, the discovery of small molecule inhibitors has attracted significant attention in the scientific community. Efforts to discover SHP2-targeted small molecule inhibitors targeting the active site such as NSC87877,^10^ II-B08,^11^ and NAT6-297775^12^ provide insights into the inhibitory mechanism that helps to design more efficient inhibitors. However, these active site inhibitors often lack selectivity because of high protein sequence identity within the inherent catalytic site among all PTPs. Additionally, the solvated, polar, and positive-charged environment of the PTP active pocket requires multiple ionisable functional groups of inhibitors to compete with the substrate. These functional groups, however, cause problems of low potency, poor cell permeability, and oral bioavailability. To overcome the obstacles of active site inhibitors, novel allosteric inhibitors stemming from chemical, mechanism-based, and computer-aided studies focussed on high selectivity and low toxicity are gaining an intensive research momentum recently. Initial medicinal chemistry research identified allosteric inhibitors targeting SHP2 with moderate potency, selectivity, and oral bioavailability, as exemplified by Novartis developed SHP099.^13^ SHP099 concurrently inserts into the “blocking” loop located at the interdomain interface of N-SH2, C-SH2, and PTP domains, which stabilises SHP2 in the inactive “closed” conformation. Moreover, SHP099 shows high selectivity over 66 kinases and 21 phosphatases including SHP1, which shares more than 60% identity with SHP2. For now, several allosteric inhibitors have been successively discovered and at least three candidates including Novartis’s TNO155,^14^ RMC-4630,^15^ and JAB-3068^16^ are currently undergoing in Phase II clinical trials. Therefore, discovery of novel allosteric inhibitors with diverse chemical backbones is of great importance.

In this study, we designed and characterised a SHP2 allosteric inhibitor, 2-((3R,4R)-4-amino-3-methyl-2-oxa-8-azaspiro[4.5]decan-8-yl)-5–(2,3-dichlorophenyl)-3-methylpyrrolo[2,1-f][1,2,4]triazin-4(3H)-one (PB17-026-01). The SHP2-inhibitor complex co-crystal structure was studied, which provides more detailed information about the binding pattern of PB17-026-01 to SHP2 and the stabilised auto-inhibited conformation of SHP2. In summary, our results provide new insights into the interaction mode of SHP2 allosteric inhibitor with SHP2 and a guide for the design of SHP2 allosteric inhibitor.

## Results and discussion

**Figure 1.**
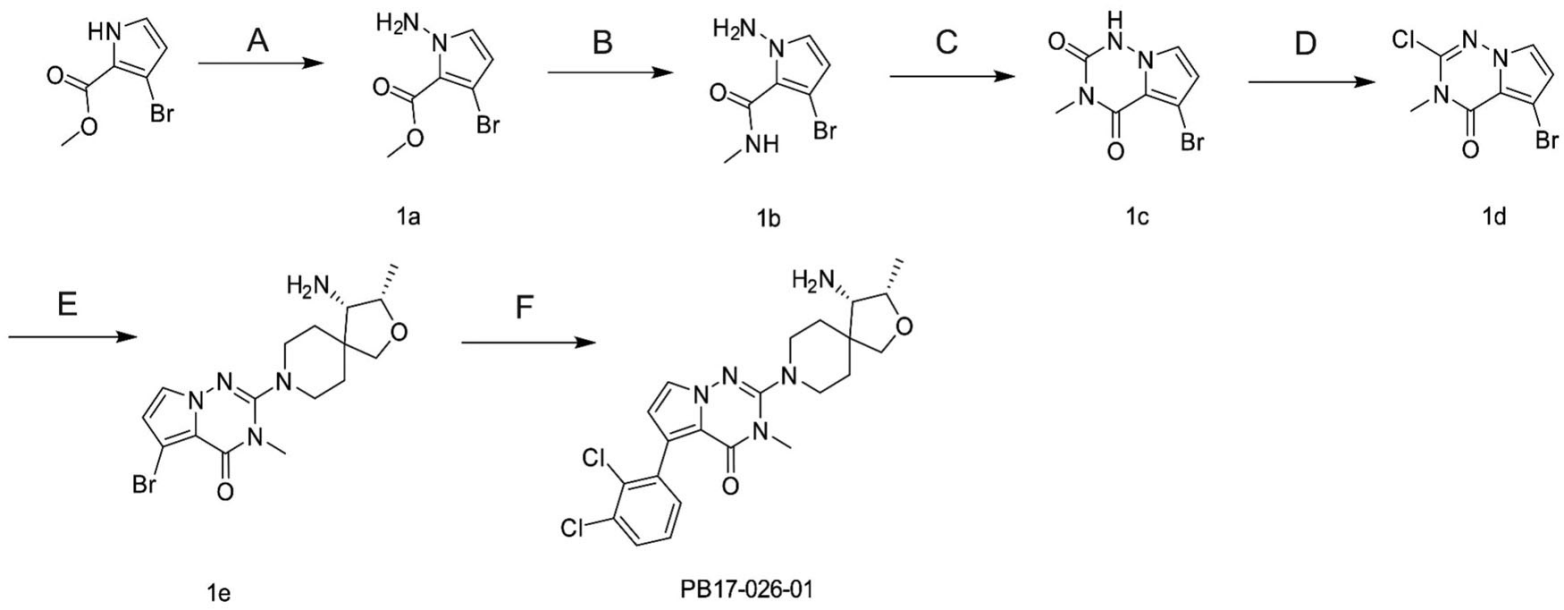
The synthesis of PB17-026-01. Reagents and conditions: (A) NaH, O-diphenylphosphorylhydroxylamine, DMF, RT, 2 h, 85% yield; (B) methanol ammonia, MeOH, 100 °C, 10 h, 79.1% yield; (C) NaOH, triphosgene, THF, 76.2% yield; (D) POCl_3_, DIPEA, 100 °C, 10 h, 66% yield; (E) NMP, triisopropanolamine, 100 °C, 2 h, 65.6% yield; and (F) 2,3-dichlorophenylboronic acid, K_3_PO_4_, dioxane, Pd(dppf)Cl_2_, 90 °C, 10 h, 69.6% yield.

**Figure 2.**
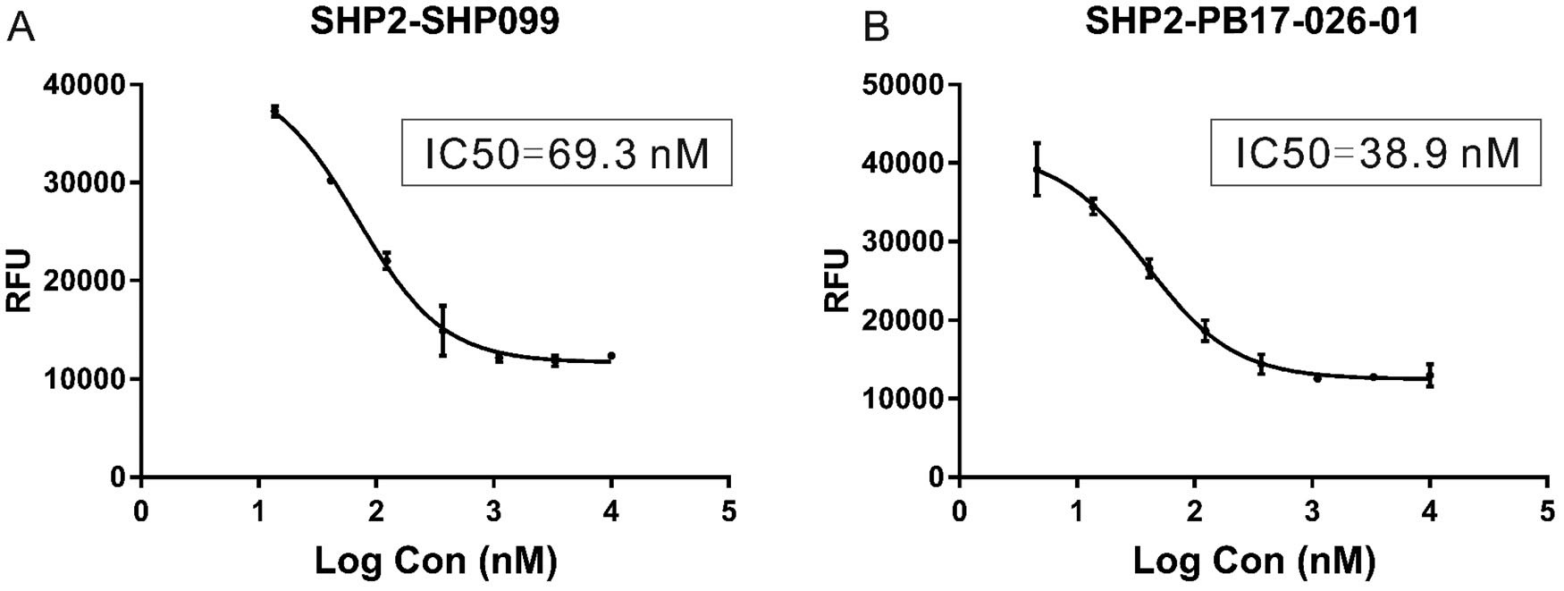
IC_50_ (half maximal inhibitory concentration) of inhibitors determined by dose–response assay for SHP2. (A) Dose–inhibition curve of SHP099. (B) Dose–inhibition curve of PB17-026-01.

We first designed a series of compounds (see supporting information for the spectral data including ^1^H-NMR and ^13^C-NMR about the compounds) derived from SHP099 and their inhibitory activities were measured (Table 1 and Figure 1). Of these compounds, the compound PB17-026-01 showed the highest inhibitory activity with a IC_50_ value of 38.9 nM, which is better than SHP099 (Figure 2).

**Table 1.** Chemical structures and inhibitory activities of different compounds.

ID	Structure	Inhibitory activity IC_50_ (nM)
PB17-021-01	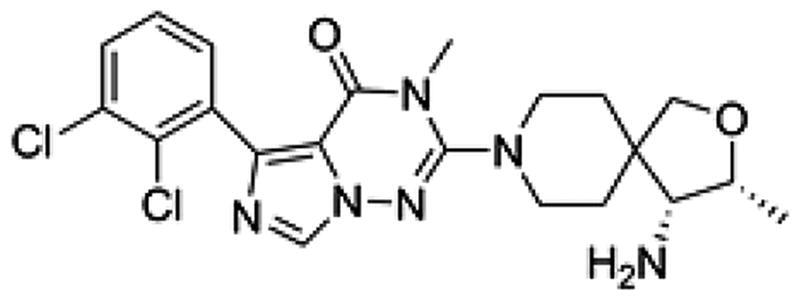	104.2
PB17-026-01	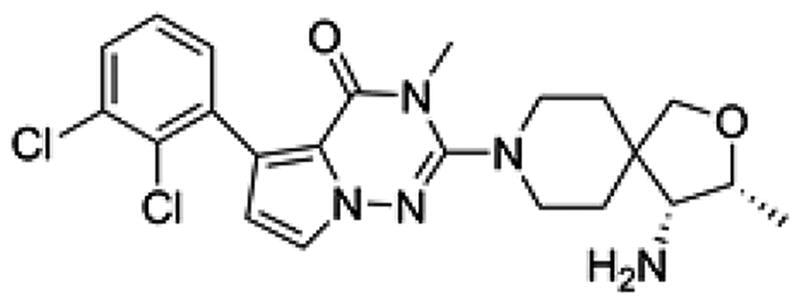	38.9
PB17-035-01	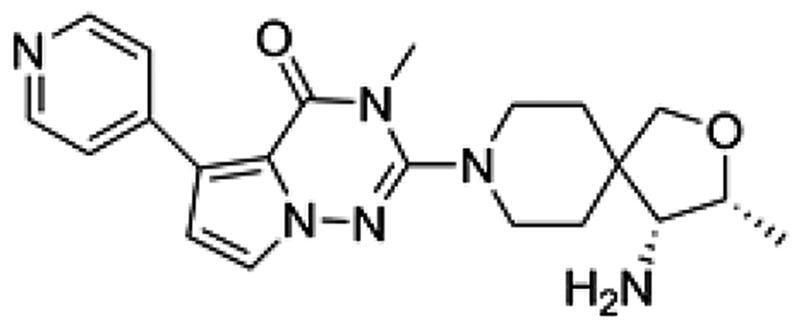	168
PB17-036-01	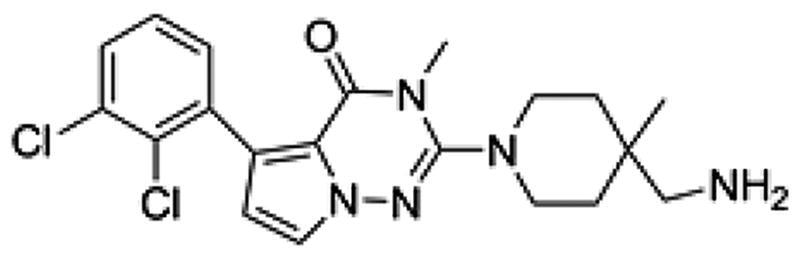	645
SHP099	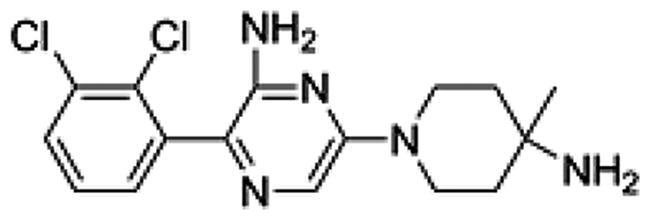	69.3

**Figure 3.**
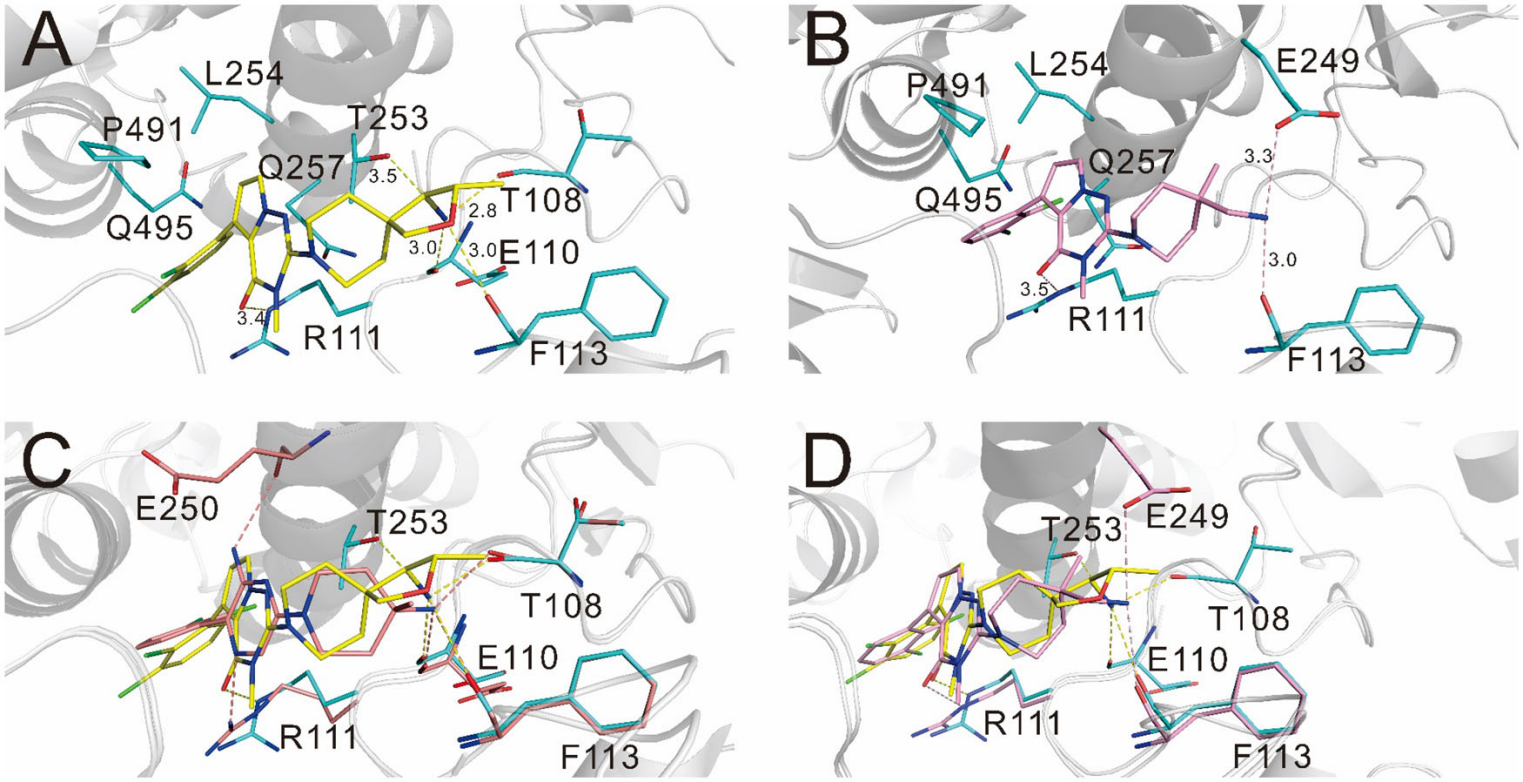
(A) Interactions between PB17-026-01 and SHP2. (B) Interactions between PB17-036-01 and SHP2. (C) Superposition of the co-crystal structures of SHP2 in complex of SHP099 (salmon) and PB17-026-01 (yellow). Compared with SHP099, PB17-026-01 lacks the hydrogen bond with E250 but forms hydrogen bond with T253, meanwhile it is hydrogen-bonded with N_ε_ instead of N_η_ of R111. (D) Superposition of the co-crystal structures of SHP2 in the complex of PB17-036-01 (pink) and PB17-026-01 (yellow). The terminal group of PB17-026-01 is hydrogen-bonded with four residues T108, E110, F113, and T253, whereas the terminal group of PB17-036-01 is only hydrogen-bonded with F113 and E249.

To investigate how PB17-026-01 interacts with SHP2, we studied the co-crystal structure of SHP2 in complex with PB17-026-01 (PDB-ID: 7XBQ) (see Tables 2 and 3 for detailed information about protein expression and crystallised macromolecule preparation). The crystal structure solved at 2.20 Å resolution provides detailed information about the interaction mode between SHP2 and PB17-026-01. The crystal belongs to the orthorhombic space group P2_1_2_1_2_1_ with the unit cell dimensions of a = 55.69 Å, b = 91.39 Å, and c = 212.98 Å (Table 4). As previously reported, the SHP2 protein contains three potential small molecule modalities: (1) the “tunnel-like” pocket formed by the inter-domain interface of N-SH2, C-SH2, and PTP domains; (2) the “latch” locus located at the interface of N-SH2 and PTP domains, which is approximately 20 Å from the tunnel; (3) the “groove” site on the opposite side of the protein.^17^ The electron density clearly reveals that PB17-026-01 perfectly adheres to the central tunnel region (estimated volume = 464 Å^3^) and interacts with all three domains of SHP2, locking the enzyme in an auto-inhibited, inactive conformation (Supplementary Figure S1A). At the tunnel interface, PB17-026-01 is hydrogen-bonded with N_ε_ of R111 in the N-SH2 domain, main chain carboxyl O of F113 in the C-SH2 domain, O_β_ of T253 in the PTP domain, main chain carboxyl O of T108 and E110, respectively (Figure 3(A)). In addition, the dichlorophenyl moiety of PB17-026-01 occupies an extensive hydrophobic area of the pharmacophore by interacting with L254, Q257, P491, and Q495 in the PTP domain. Compared with SHP099 (Supplementary Figure S2), PB17-026-01 lacks the hydrogen bond with E250 but forms hydrogen bond with T253; meanwhile it is hydrogen-bonded with N_ε_ instead of N_η_ of R111(Figure 3(C)). The binding of PB17-026-01 does not change the conformation of SHP2 significantly and the co-crystal structure is almost identical with the inactive apo SHP2 (PDB code: 2SHP) with the N-SH2 domain sealing the active site. The interaction between the N-SH2 domain and the PTP domain remains in resting state with unchanged relative position of each domain. PB17-021-01 differs from PB17-026-01 only by a replacement of a C atom with a N atom on the five-member ring; however, the former has a decreased inhibition activity (IC_50_ = 104.2 nM). Co-crystal structure of SHP2 in complex with PB17-026-01 shows that this C atom is in the vicinity of two hydrophobic residues L254 and P491. There are inherent hydrophobic and desolvation penalties incurred upon the substitution of a C atom with a N atom which leads to the decreased binding activity.

**Table 2.** Macromolecule production information.

Source organism	*Homo sapiens*
DNA source	*Homo sapiens*
Forward primer	ATGACATCGCGGAGATGGTT
Reverse primer	CTATAGTGTTTCAATATAAT
Cloning vector	pET-15b
Expression vector	pET-15b
Expression host	*E. coli* BL21 (DE3)
Complete amino acid sequence of the construct produced	MTSRRWFHPNITGVEAENLLLTRGVDGSFLARPSKSNPGDFTLSVRRNGAVTHIKIQNTG DYYDLYGGEKFATLAELVQYYMEHHGQLKEKNGDVIELKYPLNCADPTSERWFHGHLSGK EAEKLLTEKGKHGSFLVRESQSHPGDFVLSVRTGDDKGESNDGKSKVTHVMIRCQELKYD VGGGERFDSLTDLVEHYKKNPMVETLGTVLQLKQPLNTTRINAAEIESRVRELSKLAETT DKVKQGFWEEFETLQQQECKLLYSRKEGQRQENKNKNRYKNILPFDHTRVVLHDGDPNEP VSDYINANIIMPEFETKCNNSKPKKSYIATQGCLQNTVNDFWRMVFQENSRVIVMTTKEV ERGKSKCVKYWPDEYALKEYGVMRVRNVKESAAHDYTLRELKLSKVGQGNTERTVWQYHF RTWPDHGVPSDPGGVLDFLEEVHHKQESIMDAGPVVVHCSAGIGRTGTFIVIDILIDIIR EKGVDCDIDVPKTIQMVRSQRSGMVQTEAQYRFIYMAVQHYIETL

**Table 3.** Crystallisation.

Method	Sitting drop vapour diffusion
Plate type	MRC 96-well two-drop sitting drop plates
Temperature (K)	297
Protein concentration	12 mg/mL
Buffer composition of protein solution	5 mM Tris-HCl, pH 8.0, 100 mM NaCl, and 5 mM DTT
Composition of reservoir solution	13–18% PEG 4000, and 0.1 M Tris-HCl, pH 8.5
Volume and ratio of drop	1.0 μL 1:1
Volume of reservoir	50 μL

**Table 4.** Data collection statistics for crystals of PB17-026-01 and SHP2 complexes, PB17-036-01 and SHP2 complexes.

Diffraction source	The Shanghai Synchrotron Radiation Facility beamline BL19U1	The Shanghai Synchrotron Radiation Facility beamline BL18U1
Wavelength (Å)	0.9793	0.9793
Temperature (K)	100	100
Detector	DECTRIS PILATUS 6 M	DECTRIS PILATUS 6 M
Crystal-detector distance (mm)	200	350
Rotation range per image (°)	1	0.5
Total rotation range (°)	360	180
Exposure time per image (s)	0.5	0.25
Space group	P 2_1_ 2_1_ 2_1_	P 2_1_ 2_1_ 2_1_
*a*, *b*, *c* (Å)	55.69, 91.39, 212.98,	56.4995, 91.1698, 213.57,
α, β, γ (°)	90.00, 90.00, 90.00	90.00, 90.00, 90.00
Mosaicity (°)	0.68	0.56
Resolution range (Å)	39.51–2.20 (2.26–2.20)	38.81–3.0 (3.107–3.0)
Total no. of reflections	286,890	45,174 (4395)
No. of unique reflections	56,019	22,767 (2223)
Completeness (%)	99.6 (99.7)	92.87 (85.46)
Redundancy	5.1 (5.1)	2.0 (2.0)
〈*I*/σ(*I*)〉	**4.6 (1.4) ^#^**	**5.85 (2.90)** ^#^
*R*_r.i.m._	**0.176 (1.123)**	**0.068 (0.1992)**
Overall *B* factor from Wilson plot (Å^2^)	**30.3**	**36.45**

^#^ Numbers in parentheses represent the value for the highest resolution shell.

PB17-036-01 is ∼20-fold less active (IC_50_ = 645 nM) than PB17-026-01and the only difference between the two compounds is at the terminal group. To investigate the structure–activity relationship, we examined the co-crystal structure of SHP2 in complex with PB17-036-01 (PDB-ID: 8GWW). The crystal structure was solved at 3.0 Å resolution and the structure clearly reveals that PB17-036-01 perfectly adheres to the central tunnel region resembling PB17-026-01 (Supplementary Figure S1B). Superimposition of the co-crystal structures of PB17-036-01 and the lead inhibitor PB17-026-01 (Figure 3(D)) show that PB17-036-01 is hydrogen-bonded with N_ε_ of R111 in the N-SH2 domain, main chain carboxyl O of F113, and side-chain carboxyl O of E249 in the same mode as PB17-026-01 (Figure 3(B)). However, the terminal group of PB17-026-01 is hydrogen-bonded with four residues T108, E110, F113, and T253, whereas the terminal group of PB17-036-01 is only hydrogen-bonded with F113 and E249, which explains the improved binding activity of PB17-026-01.

## Conclusion

As the central role of SHP2 in developmental and oncogenic diseases, the development of potent, selective, and orally efficacious SHP2 inhibitors has attracted interest of many pharmaceutical researches and developments but remains a challenge. In this study, we identified a new compound PB17-026-01 derived from SHP099 but with significantly different chemical backbone that allosterically inhibits SHP2 potently. We also solved the co-crystal structures of SHP2 in complex with PB17-026-01 and its analogue compound PB17-036-01, which only differs from PB17-026-01 at the terminal group. These structures indicate that both of PB17-026-01 and PB17-036-01 are inserted in the tunnel pocket to stabilise the auto-inhibited and inactive conformation with different binding mode form SHP099 and PB17-026-01 forms more hydrogen bonds with SHP2 than PB17-036-01 because of their different terminal groups. For its high potency and new backbone structure, the compound provides new insights into SHP2 inhibition and a suitable basis for further development of potent, selective, and orally bioavailable compounds leading to treat SHP2-dependent diseases.

## Experimental section

### The synthesis of PB17-026-01

A small amount of sodium hydride (NaH, 130 mM) was added to methyl 3-bromopyrrole-2-carboxylate (100 mM) in DMF (100 mL) multiple times. The reaction mixture was stirred at 0 °C for 20 min, followed by the addition of the O-diphenylphosphinylhydroxylamine (120 mM). The mixture was incubated at room temperature for 2 h and extracted twice with ethyl acetate. The organic phase was washed, dried over water, and concentrated. The residue was purified by column chromatography to afford intermediate 1a as a white solid (17.0 g, 85% yield). Liquid chromatography–mass spectrometry (LC–MS) (m/z): 218.0/220.0 [M + H]^+^ (Figure 1).

Methanol ammonia (2 M, 30 mL) was added to intermediate 1a (64 mM) in methanol (50 mL). The reaction was heated to 100 °C for 10 h. After cooling, the organic phase was removed, and intermediate 1b (11.0 g) was separated and purified by column chromatography, with a yield of 79.1%. LC–MS (m/z): 217/219.0 [M + H]^+^.

Sodium hydroxide (100 mM) and triphosgene (50 mM) was added to intermediate 1b (50 mM) in tetrahydrofuran (60 mL). A large amount of solid were precipitated, filtered, washed with dichloromethane, and dried to obtain intermediate 1c (9.3 g, 76.2% yield). LC–MS (m/z): 243/245.0 [M + H]^+^.

A catalytic amount of DIPEA was added to intermediate 1c (5.0 g, 20 mM) in phosphorus trichloride (10 mL) and heated to 100 °C for 10 h. When the reaction was finished, phosphorus trichloride was removed by distillation, and intermediate 1d (3.6 g) was obtained by column chromatography in 66.0% yield. LC–MS (m/z): 261/263.0 [M + H]^+^. 1H NMR (400 MHz, DMSO) δ 7.65 (d, J = 2.9 Hz, 1H), 6.73 (d, J = 2.9 Hz, 1H), 3.45 (s, 3H).

Intermediate 1d (1 mM) and (3S,4S)-3-methyl-2-oxa-8-azaspiro[4.5]decan-4-amine dihydrochloride (2 mM) were dissolved in 5 mL of N-methylpyrrolidone, while triisopropylamine (2 mL) was added and the reaction was heated to 100 °C for 2 h. Intermediate 1e (260 mg) was obtained by column chromatography in 65.6% yield. LC–MS (m/z): 395.0/397.0 [M + H]^+^.

A mixture of the intermediate 1e (200 mg, 0.5 mM), 2,3-dichlorophenylboronic acid (380 mg, 1 mM), 2 M potassium phosphate (0.75 mL) in dioxane (20 mL), and Pd(dppf)Cl_2_ (0.1 eq) was stirred at 90 °C for 10 h. The product was added anhydrous ethanol (50 mL) and purified by column chromatography to obtain 160 mg of the final product in 69.6% yield. LC–MS (m/z): 462.1 [M + H]^+^. 1H NMR (400 MHz, DMSO) δ 7.61 (dd, J = 6.3, 3.3 Hz, 1H), 7.52 (d, J = 2.7 Hz, 1H), 7.38–7.32 (m, 2H), 6.57 (d, J = 2.7 Hz, 1H), 4.16–4.08 (m, 1H), 3.73 (d, J = 8.7 Hz, 1H), 3.55 (d, J = 8.7 Hz, 1H), 3.33 (s, 3H), 3.23 (d, J = 18.5 Hz, 2H), 3.11 (d, J = 4.7 Hz, 1H), 2.95–2.79 (m, 2H), 1.93–1.75 (m, 2H), 1.63 (dd, J = 31.2, 13.3 Hz, 2H), 1.14 (d, J = 6.4 Hz, 3H).

### Protein expression and purification

The gene sequence encoding SHP2 residues Met1-Leu525 (UniProt accession code Q06124) was synthesised by GenScript (GenScript.com) with codon optimisation for *E. coli* expression and inserted into pET-15b containing an N-Terminal 6 × His-tag and a tobacco etch virus (TEV) cleavage site. The *E. coli* BL21 (DE3) cells transformed with the recombinant plasmid were grown in Luria-Bertani medium at 37 °C until OD_600_ reached 0.6–0.8. Protein expression was induced by isopropyl-β-D-thiogalactoside to a final concentration of 0.2 mM and the cells were grown overnight at 18 °C and then harvested by centrifugation at 6000*g* for 15 min at 4 °C. The cell pellet was re-suspended in buffer A (50 mM Tris-HCl, pH 8.0, 500 mM NaCl, 5% glycerol) and lysed by a high-pressure homogeniser at 1000 bar. Cell debris was removed by centrifugation at 47,000*g* for 30 min. The supernatant was loaded onto a 5 mL Ni-NTA column (GE Healthcare), which had been equilibrated in buffer A. A gradient wash was applied with increasing concentration of imidazole (10–50 mM) in the same buffer in order to remove non-specific bound protein. The His-tagged SHP2 was eluted with 250 mM imidazole in the same buffer. The 6 × His-tag was cleaved by TEV protease overnight at 4 °C. The TEV protease and uncleaved 6 × His-tagged protein were removed by passing through the Ni-NTA column again. The flow-through containing His-tag removed SHP2 was concentrated and further purified by a size-exclusion column Superose 12 10/300 (GE Healthcare) and the purest fraction of SHP2 was concentrated to ∼12 mg/mL in 5 mM Tris-HCl, pH 8.0, 100 mM NaCl, and 5 mM DTT.

### Crystallisation and structure determination

Crystallisation was performed in MRC 96-well two-drop sitting drop plates by using the sitting-drop vapour-diffusion method. Half microliter of protein solution containing 12 mg/mL in 5 mM Tris-HCl pH 8.0, 100 mM NaCl, and 5 mM DTT was mixed with 0.5 μL of crystallisation well solution containing 13–18% PEG 4000, and 0.1 M Tris-HCl pH 8.5. The crystals appeared after a few hours and reached the maximum size after 24 h. Then the crystals were transferred to a new drop containing 18% PEG 4000, and 0.1 M Tris-HCl pH 8.5 and the inhibitor powders were added to the drop to soak the crystals. The SHP2-inhibitor complex crystals were transferred to a cryoprotectant solution comprising 18% PEG 4000, 0.1 M Tris-HCl pH 8.5 and 20% glycerol, and then flash frozen in liquid nitrogen. Diffraction data were collected from a single crystal at 100 K at BL19U1 of the Shanghai Synchrotron Radiation Facility. The reflections were indexed, integrated, and processed with iMosflm.^18^ The space group of the complex was P2_1_2_1_2_1_ with two molecules in the asymmetric unit. The structure was solved by molecular replacement implemented in Phaser^19^ using the SHP2 structure (PDB code 6WU8) as the original search model. The structure model of the inhibitor was manually fit in the electron density using Coot.^20^ Structure refinements were performed by using Phenix^21^ (Table 5) and figures of the models were created with PYMOL (The PyMOL Molecular Graphics System, Schrödinger, LLC).

**Table 5.** Structure solution and refinement. Values for the outer shell are given in parentheses.

Crystal	SHP2 in complex with PB17-026-01	SHP2 in complex with PB17-036-01
Completeness (%)	99.6 (99.7)	92.87 (85.46)
σ cut-off	1.4	
No. of reflections, working set	55,948 (5515)	21,269 (1910)
No. of reflections, test set	1999 (197)	1863 (162)
Final *R*_cryst_	0.2401 (0.3346)	0.2398 (0.3089)
Final *R*_free_	0.2746 (0.3466)	0.2876 (0.3977)
Cruickshank DPI		
No. of non-H atoms	7664	7386
Protein	7132	7330
Ion	0	0
Ligand	62	56
Water	470	0
Total	7664	7386
RMS deviations		
Bonds (Å)	0.003	0.002
Angles (°)	0.60	0.40
Average *B* factors (Å^2^)	34.89	34.89
Protein	34.58	32.54
Ion		
Ligand	33.49	37.35
Water	39.79	0
Ramachandran plot		
Most favoured (%)	96.50	95.56
Allowed (%)	3.06	4.23

### Measurement of phosphatase activity

The auto-inhibition conformation of SHP2 is allosterically activated by the binding of bis-tyrosyl-phosphorylated peptides, which makes the PTP domain of SHP2 active and available for substrate recognition and subsequent reaction catalysis. The measurement of the catalytic activity of SHP2 is using the surrogate substrate 6,8-difluoro-4-methylumbelliferyl phosphate (DiFMUP) in a prompt fluorescence assay format.^14^^,^^22^^,^^23^ In special, the phosphatase reaction was performed at room temperature in a 384-well black plate (Corning) with a total volume of 20 μL. The assay buffer contained 60 mM HEPES, pH7.2, 75 mM NaCl, 75 mM KCl, 1 mM EDTA, 5 mM DTT, and 0.05% Triton X-100.

The inhibition of SHP2 from the 5 μL tested compound was monitored using an assay in which 0.5 nM of SHP2 was incubated with of 0.125 µM of bis-tyrosyl-phosphorylated peptide. After 60 min incubation at room temperature, the surrogate substrate DiFMUP (Thermo, Cat: D6567, 200 μM) was added to the catalytic reaction. After 30 min incubation at room temperature, the fluorescence signal was measured at the excitation and emission wavelengths of 340 nm and 450 nm respectively with a plate reader (Tecan Spark). The curves of the inhibitor dose–response were analysed using normalised IC_50_ regression curve fitting with control-based normalisation.

## Supplementary Material

Supplemental Material
