# Supplementary material for: Discovery of the SHP2 allosteric inhibitor 2-((3R,4R)-4-amino-3-methyl-2-oxa-8-azaspiro[4.5]decan-8-yl)-5-(2,3-dichlorophenyl)-3-methylpyrrolo[2,1-f][1,2,4] triazin-4(3H)-one
Source: J Enzyme Inhib Med Chem. 2022 Dec 8;38(1):398–404. doi: 10.1080/14756366.2022.2151594 (PMC9744210; doi:10.1080/14756366.2022.2151594)
Supplement: Supplemental Material [file IENZ_A_2151594_SM3804.pdf]

Discovery of the SHP2 allosteric inhibitor 2-((3R,4R)-4-amino-3-methyl-2-oxa-8-azaspiro[4.5]decan-8-yl)-5-(2,3-dichlorophenyl)-3-methylpyrrolo[2,1-f][1,2,4]triazin-4(3H)-one

Yanmei Luo[a#], Jin Li[b#], Yuliang Zong[a], Mengxin Sun[a], Wan Zheng[a], Jiapeng Zhu[a, c], Liu Liu[b*] and Bing Liu[a, c*]

[a]*School of Medicine, Nanjing University of Chinese Medicine, Nanjing, China;*

[b] *Division of Medicinal Chemistry, PharmaBlock Sciences (Nanjing), Inc., Nanjing, China.*

[c]*Jiangsu Key Laboratory for Pharmacology and Safety Evaluation of Chinese Materia Medica, School of Pharmacy, Nanjing University of Chinese Medicine, Nanjing, China*

Correspondence email: bingliu@njucm.edu.cn, liu_liu@PharmaBlock.com

#These authors contributed equally.

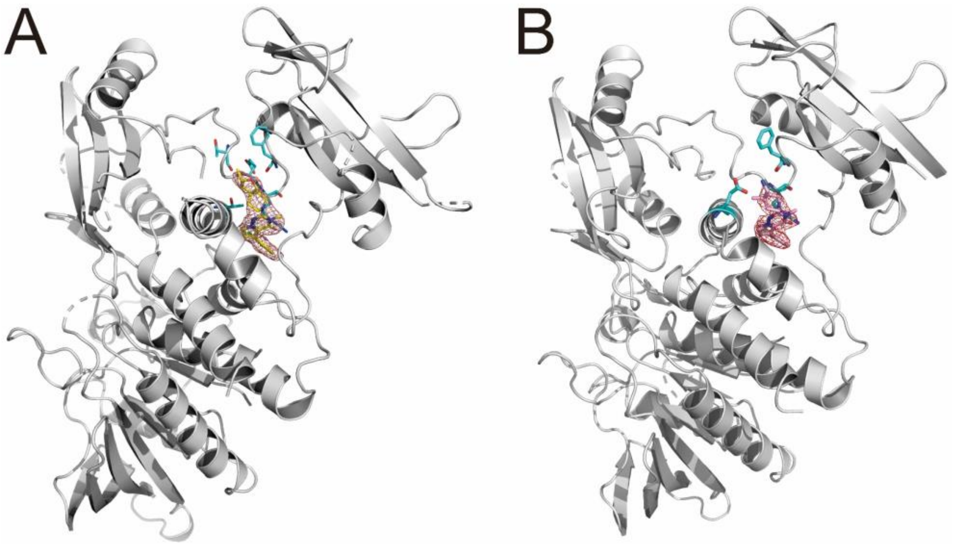

Figure S1: Crystal structure of SHP2 inhibited by (A) PB17-026-01 at 2.20 Å resolution and (B) PB17-036-01 at 3.0 Å resolution. They are located in the allosteric binding site at the junction of three domains.

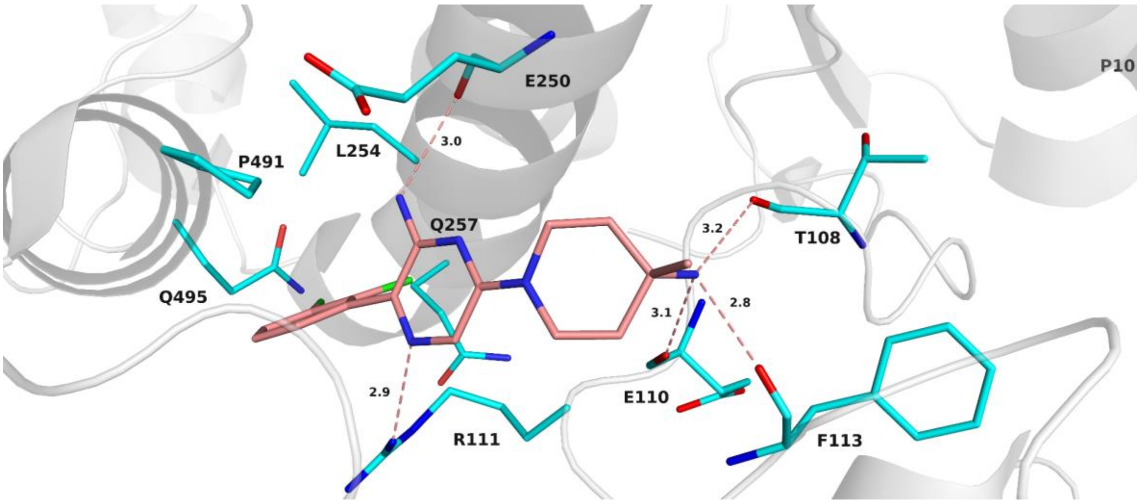

Figure S2: Interactions between SHP099 and SHP2.

The ^{1}H-NMR and ^{13}C-NMR of the compounds.

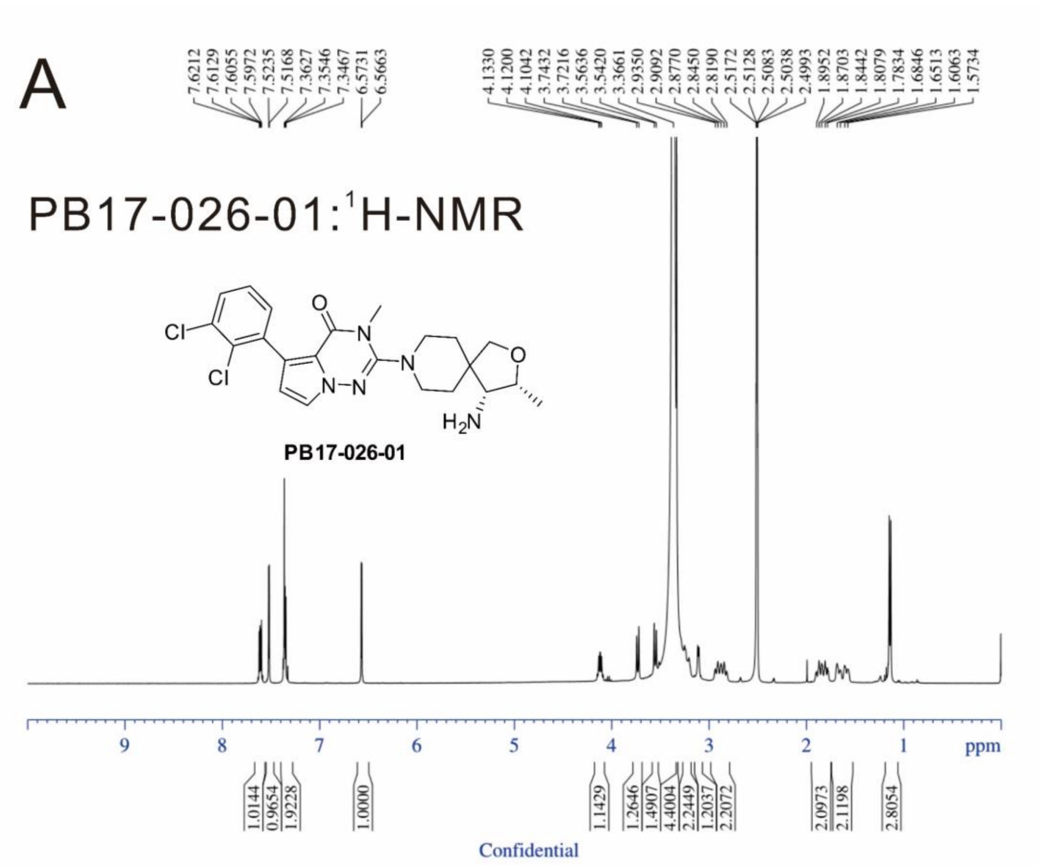

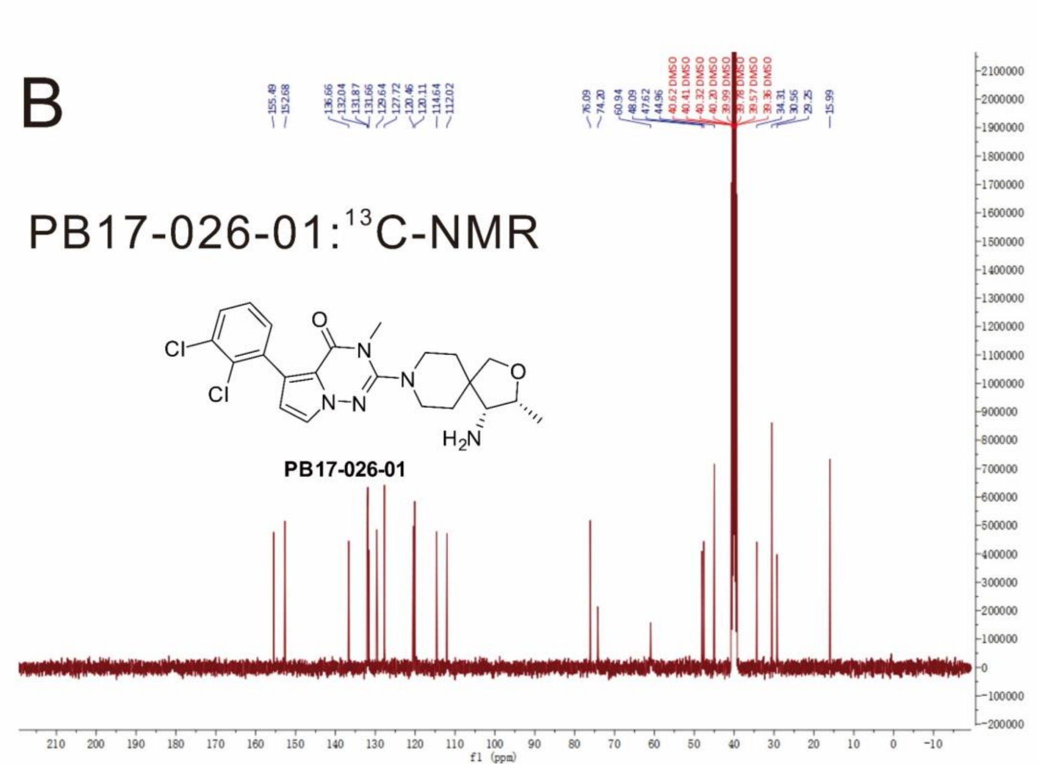

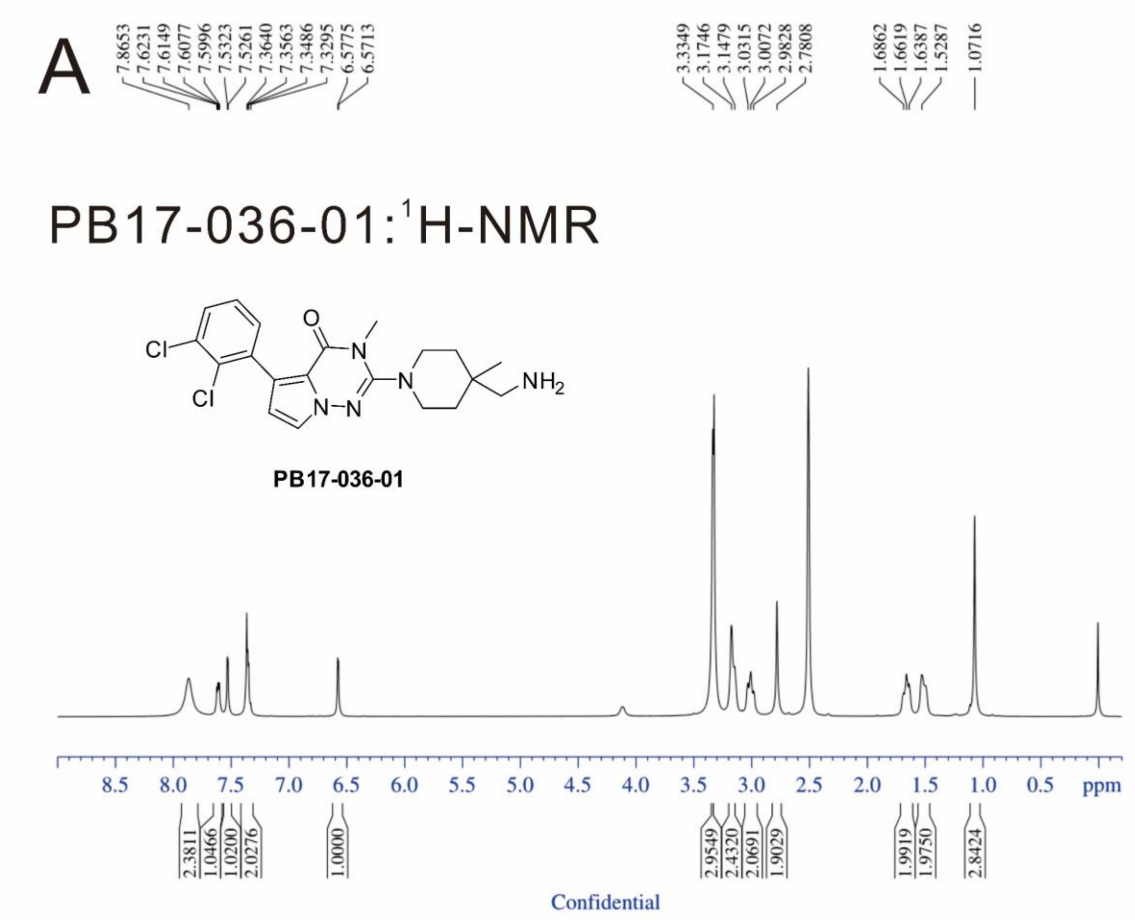
A
7.8653
7.6231
7.6149
7.6077
7.5996
7.5323
7.5261
7.3640
7.3563
7.3486
7.3295
6.5775
6.5713
3.3349
3.1746
3.1479
3.0315
3.0072
2.9828
2.7808
1.6862
1.6619
1.6387
1.5287
1.0716
PB17-036-01:1H-NMR
Cl
Cl
O
N
N
N
N
N
NH2
PB17-036-01
8.5 8.0 7.5 7.0 6.5 6.0 5.5 5.0 4.5 4.0 3.5 3.0 2.5 2.0 1.5 1.0 0.5 ppm
2.3811
1.0466
1.0200
2.0276
1.0000
2.9549
2.4320
2.0691
1.9029
1.9919
1.9750
2.8424
Confidential

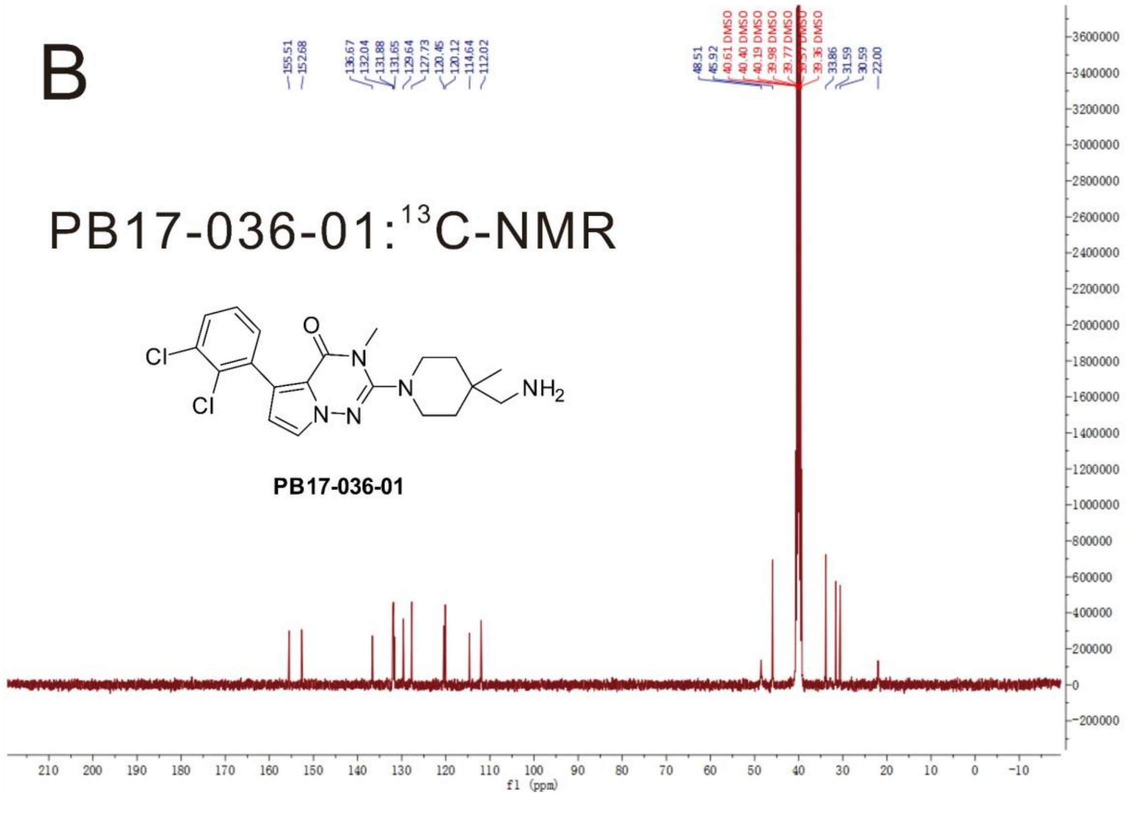
B
155.51
152.68
136.67
132.04
131.88
131.65
129.64
127.73
120.45
120.12
114.64
112.02
48.51
45.92
40.61 DMSO
40.40 DMSO
40.19 DMSO
39.98 DMSO
39.77 DMSO
39.57 DMSO
39.36 DMSO
33.86
31.59
30.59
22.00
PB17-036-01:13C-NMR
Cl
Cl
O
N
N
N
N
N
NH2
PB17-036-01
210 200 190 180 170 160 150 140 130 120 110 100 90 80 70 60 50 40 30 20 10 0 -10
f1 (ppm)

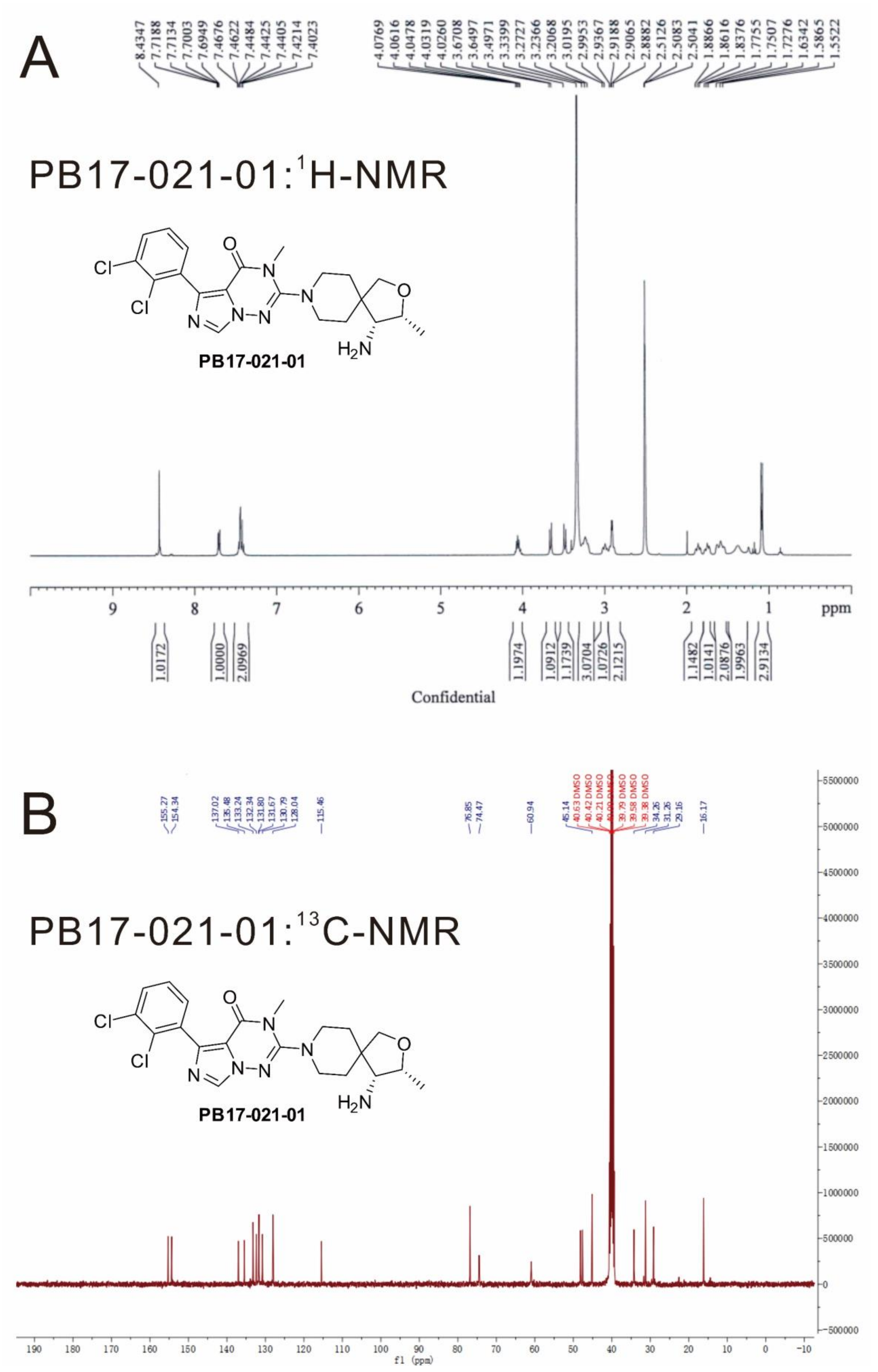
A
PB17-021-01:1H-NMR
PB17-021-01
8.4347
7.7188
7.7134
7.7003
7.6949
7.4676
7.4622
7.4484
7.4425
7.4405
7.4214
7.4023
4.0769
4.0616
4.0478
4.0319
4.0260
3.6708
3.6497
3.4971
3.3399
3.2727
3.2366
3.2068
3.0195
2.9953
2.9367
2.9188
2.9065
2.8882
2.5126
2.5083
2.5041
1.8866
1.8616
1.8376
1.7755
1.7507
1.7276
1.6342
1.5865
1.5522
ppm
1.0172
1.0000
2.0969
1.1974
1.0912
1.1739
3.0704
1.0726
2.1215
1.1482
1.0141
2.0876
1.9963
2.9134
Confidential
B
PB17-021-01:13C-NMR
PB17-021-01
155.27
154.34
137.02
135.48
133.24
132.34
131.80
131.67
130.79
128.04
115.46
76.85
74.47
60.94
45.14
40.63 DMSO
40.42 DMSO
40.21 DMSO
39.79 DMSO
39.58 DMSO
39.38 DMSO
34.26
31.26
29.16
16.17
f1 (ppm)

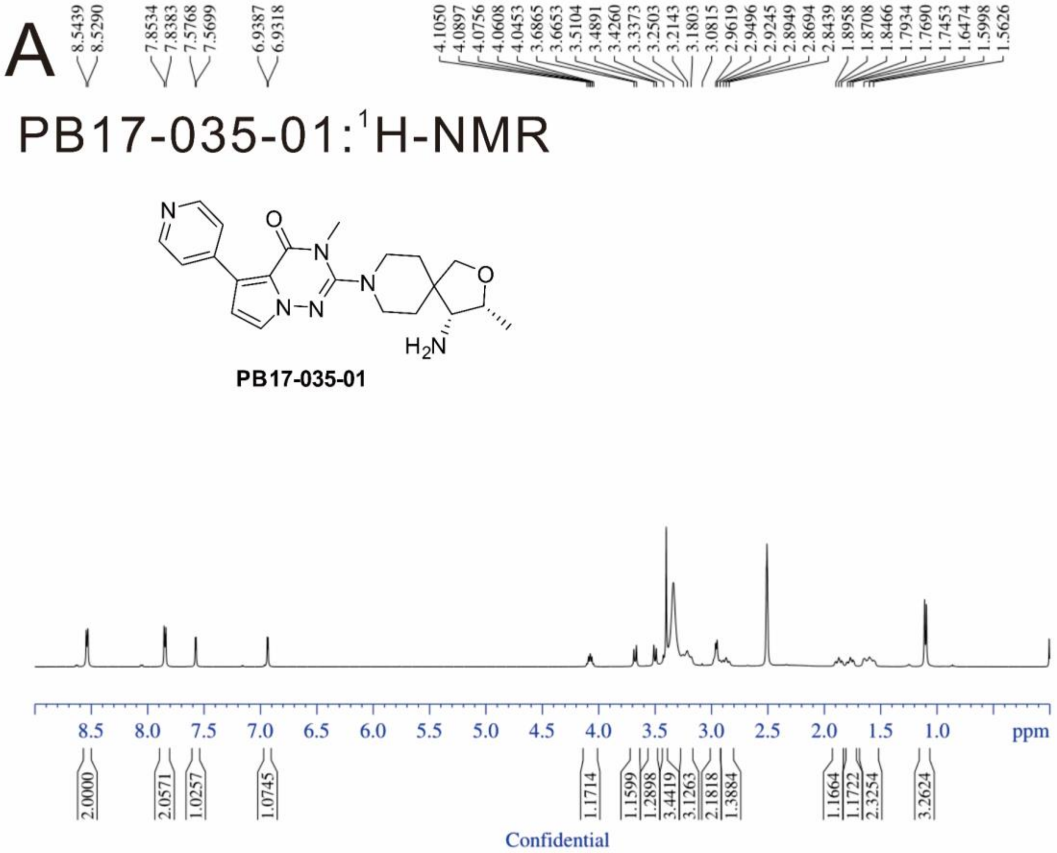
A
PB17-035-01:1H-NMR
8.5439
8.5290
7.8534
7.8383
7.5768
7.5699
6.9387
6.9318
4.1050
4.0897
4.0756
4.0608
4.0453
3.6865
3.6653
3.5104
3.4891
3.4260
3.3373
3.2503
3.2143
3.1803
3.0815
2.9619
2.9496
2.9245
2.8949
2.8694
2.8439
1.8958
1.8708
1.8466
1.7934
1.7690
1.7453
1.6474
1.5998
1.5626
PB17-035-01
ppm
2.0000
2.0571
1.0257
1.0745
1.1714
1.1599
1.2898
3.4419
3.1263
2.1818
1.3884
1.1664
1.1722
2.3254
3.2624
Confidential

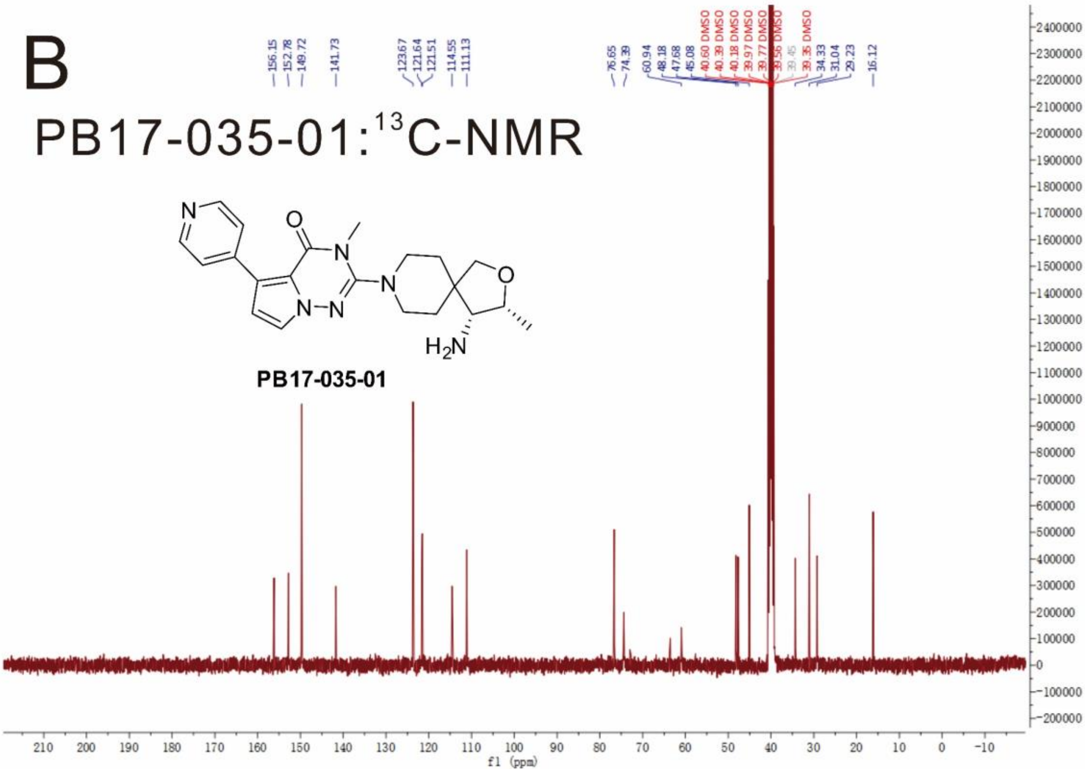
B
PB17-035-01:13C-NMR
156.15
152.78
149.72
141.73
123.67
121.64
121.51
114.55
111.13
76.65
74.39
60.94
48.18
47.68
45.08
40.60 DMSO
40.39 DMSO
40.18 DMSO
39.97 DMSO
39.77 DMSO
39.56 DMSO
39.45
39.35 DMSO
34.33
31.04
29.23
16.12
PB17-035-01
f1 (ppm)
